# General and disease-specific quality of life in patients with chronic suppurative otitis media - a prospective study

**DOI:** 10.1186/1477-7525-9-48

**Published:** 2011-06-29

**Authors:** Ingo Baumann, Bianca Gerendas, Peter K Plinkert, Mark Praetorius

**Affiliations:** 1 Department of Otorhinolaryngology, Head and Neck Surgery, Medical Center of the University of Heidelberg, Im Neuenheimer Feld 400, 69120 Heidelberg, Germany; 2 Division of Otology and Neurotology, Department of Otorhinolaryngology, Head and Neck Surgery, Medical Center of the University of Heidelberg, Im Neuenheimer Feld 400, 69120 Heidelberg, Germany

## Abstract

**Background:**

Chronic suppurative otitis media (CSOM) is frequently associated with symptoms of inflammation like discharge from the ear or pain. In many cases, patients suffer from hearing loss causing communication problems and social withdrawal. The objective of this work was to collect prospective audiological data and data on general and disease-specific quality of life with validated quality of life measurement instruments to assess the impact of the disease on health-related quality of life (HR-QOL).

**Methods:**

121 patients were included in the study. Patients were clinically examined in the hospital before and 6 months after surgery including audiological testing. They filled in the quality of life questionnaires SF-36 and Chronic Otitis Media Outcome Test 15 (COMOT-15) pre-operatively and 6 and 12 months post-operatively, respectively.

**Results:**

Complete data records from 90 patients were available for statistical analysis. Disease-specific HR-QOL in patients with CSOM improved after tympanoplasty in all the scales of the COMOT-15. There was no difference in HR-QOL assessment between patients with mesotympanic respectively epitympanic CSOM. However, we did find the outcome to be worse in patients who received revision surgery compared with those receiving primary surgery. Audiometric findings correlated very well with the subscale hearing function from the COMOT-15 questionnaire. General HR-QOL measured with the SF-36 was not significantly changed by tympanoplasty.

**Conclusions:**

Tympanoplasty did lead to a significant improvement of disease-specific HR-QOL in patients with CSOM while general HR-QOL did not change. Very well correlations were found between the subscale hearing function from the COMOT-15 questionnaire and audiological findings. Revision surgery seems to be a predictor for a worse outcome.

## Background

Chronic suppurative otitis media (CSOM) affects approximately 2% of the population [1]. It is associated with significant functional limitations of hearing. This frequently results in communication problems impeding social interaction and professional life. In patients with severe hearing loss even a withdrawal from social activities can be observed frequently. In addition, further symptoms of CSOM such as persistent discharge from the ear, pain or frequent doctor visits may result in an impairment of the patients. In cases of cholesteatoma, which represents the most dangerous type of CSOM, complications like facial nerve paralysis, meningitis, or encephalitis may develop and potentially threaten the patient's life.

It has been demonstrated that the assessment of treatment results on the basis of functional diagnostics, survival rates, or similar parameters alone does not mirror subjective experiences of the patients. Hence, the importance of measuring subjectively assessed quality of life (QOL) is steadily increasing in clinical medicine.

This research in CSOM is only in the beginning. Nadol et al. [1] validated a questionnaire (Chronic Ear Survey, CES) using the data of 147 patients. Comparing results of the Short Form 36 Health Survey (SF-36) which could not prove significant changes of general QOL, the findings of the CES did detect significant changes of the scores as an outcome of surgical therapy. However, this questionnaire includes some single items which ask for the frequency of symptoms or medical problems but not for the subjective assessment of their severity by the patients. From our point of view, CES does not cover the subjective view of the patients adequately. Therefore, our group decided to develop and validate another disease-specific QOL questionnaire which covers subjectively assessed disease-specific QOL: the so-called Chronic Otitis Media Outcome Test 15 (COMOT-15) [2]. This study presents the first prospectively collected data using the COMOT-15.

## Methods

We present the data of a monocentric prospective study. The study was approved by the Ethical Committee of the Faculty of Medicine at the University of Heidelberg (No. 201/2005). The study was carried out in accordance with the Declaration of Helsinki as amended in 2004. Patients gave their informed written consent before starting data collection. Patients were included in the study from April 2006 through July 2007. Data collection was completed in July, 2008.

### Patients

In this study, patients with chronic suppurative mesotympanic or epitympanic otitis media were included. For the purposes of this study, the definition of CSOM according to Bluestone [3] was inapplicable. This definition is accurate from the academic point of view but much too diversified for use in clinical studies. Therefore, inclusion in the study was determined according to Nadol [1]: disease of the middle ear and/or mastoid with irreversible mucosal damage or infection lasting more than 3 months. This definition covers adequately the clinical course and findings in our patients from a clinical point of view.

The following two main types of CSOM were differentiated:

1. chronic suppurative otitis media without cholesteatoma (chronic suppurative mesotympanic otitis media)

2. chronic suppurative otitis media with cholesteatoma (chronic suppurative epitympanic otitis media)

Inclusion criteria were: CSOM, age 18 or above and having full legal capacity. Exclusion criteria were: age below 18, loss of full legal capacity, gravidity, medical or surgical treatments or conditions having the potential to influence the outcome of the study.

### Methods

Patients who were treated at the Department of Otolaryngology at the University of Heidelberg fulfilling the inclusion criteria were asked to participate in the study. Data collection was performed prospectively at three times of measurement (TM): pre-operatively (TM1), 6 months after surgery (TM2), and 12 months after surgery (TM3).

Tympanoplasty was performed in all patients. In most of the cases a retroauricular incision with a tympanomeatal flap was made. In cholesteatoma cases canal wall up and canal wall down procedures were performed according to the extension of the disease. For reconstruction of the tympanic membrane we used temporalis fascia mostly in primary surgery cases with inactive CSOM. In cases with active disease and in revision surgery compound grafts from cartilage and perichondrium or perichondrium alone harvested from the tragus were inserted. For ossicular reconstruction we used incus interpositioning or titanium-made total and partial ossicular replacement prostheses (TORP and PORP). In the latter cases a cartilage sheet of a size just a bit larger than the prosthesis head to overlap it was prepared and put on top to prevent migration of the prosthesis through the tympanic membrane.

Clinical examinations and audiologic tests were performed at TM1 and TM2. Further data (age, gender, primary or revision surgery, unilateral or bilateral disease) were collected at TM 1. Quality of life questionings were conducted at all three TM.

Clinical examination included general ENT examination, microscopy of the ears including Valsalva test and tuning fork test. In addition, the pure tone audiometry was performed. The pure tone average was measured in dB and calculated from the air conduction hearing loss at 500 Hz, 1, 2 and 4 kHz.

The QOL measurements were executed using validated measurement tools. Measurement of disease-specific QOL was performed using the Chronic Otitis Media Outcome Test 15 (COMOT-15) (Additional File 1) [2]. This instrument consists of three subscales called ear symptoms (ES, questions 1-6), hearing function (HF, questions 7-9), and mental health (MH, questions 10-13), which form the overall score (OS, questions 1-13). In addition, one question on the general evaluation of the impact of CSOM on QOL (question 14) and one question to indicate the frequency of doctor visits in the last six months as a result of CSOM (question 15) are asked. The total score and the subscores are transformed to a 0-100 scale by dividing the sum of the raw scores of the items by the sum of spans of the items followed by multiplying by 100.

The measurement of general health-related QOL life was performed using the Short Form 36 Health Survey (SF-36) [4].

The SF-36 Health Survey consists of a questionnaire with 36 items organized into several subject areas. Each item represents a scale in itself or part of a scale. The SF-36 Health Survey records eight aspects of subjective health, using different item numbers: Physical Functioning (PF, 10 items), Role-Functioning Physical (RP, 4 items), Bodily Pain (BP, 2 items), General Health (GH, 5 items), Vitality (VT, 4 items), Social Functioning (SF, 2 items), Role-Functioning Emotional (RE, 3 items), and Mental Health (MH, 5 items).

Rules for item scoring and scales are available in the SF-36 Scoring Manual. The German translation and the validation of the German translation were carried out by Bullinger and Kirchberger [5]. Evaluation was conducted by summation of the ticked item responses per scale, in doing so, for some scales a weighting was included. The scales could then be evaluated if fewer than 50 % of the items were missing. In these cases, the mean values of the existing items of a scale were used to substitute the missing items. All scales were transformed to values between 0 and 100 to allow comparisons of scales with each other and between various patient groups. Higher scores indicate a more positive rating.

Additionally and according to the scoring rules the Physical Component Score (PCS) and the Mental Component Score (MCS) were calculated.

### Statistics

Statistical evaluation was carried out using JMP^® ^version 8.0 (SAS institute Inc., Cary, NC, USA).

Standard statistical methods were used. The significance of the differences between two groups was evaluated by Student's t test. Differences within groups were tested by a paired t test. Pearson's correlation coefficient was calculated to analyze correlations of the COMOT-15 scales versus pure tone average (air conduction). The significance level for all tests was set at p <.05.

## Results

In this study 121 patients (58 males and 63 females) with a median age of 48 years (range 18-75 years) were included. Ninety patients (44 males and 46 females, response rate 74.4%) with a median age of 52 years (range 18 to 75 years) participated in all questionings and examinations. The data of these patients were used for statistical analysis. Due to the high response rate and similar gender and age distribution no response bias is to be apprehended.

The opposite (non-operated) ear in those 90 patients showed a healthy aspect in 57 cases (63%). Four patients (4%) had previously been operated on the opposite ear due to chronic suppurative mesotympanic otitis media, while 8 patients (9%) suffered from chronic suppurative mesotympanic otitis media and 7 patients (8%) suffered from cholesteatoma. No data were available for 15 patients (17%). Patients with cholesteatoma on the operated ear showed chronic suppurative mesotympanal otitis media on the opposite ear in one case and cholesteatoma in 7 cases.

### Hearing results

The tympanoplasty resulted in a significant improvement in air conduction threshold and a reduction of the air bone gap. The bone conduction threshold remained stable (Table 1).

**Table 1:** Pure tone average [dB] calculated from air conduction hearing loss [dB] at 500 Hz, 1, 2 and 4 kHz (n = 90)

	baseline [dB]	6 months [dB]	p-value (t-test)
bone conduction	24.3	22.0	0.27

air conduction	51.2	41.5	0.0035

air bone gap	25.2	17.3	< 0.0001

### COMOT-15

Both the overall score and all three subscores showed significantly better ratings for the second time of measurement, which stayed stable after 12 months except the mental health scale (Table 2).

**Table 2:** Results for the scales of COMOT-15 and SF-36 at three different times of assessment; M = mean value, SD = standard deviation, TM = time of measurement, p = p-value from Student's t-test

Questionnaire/ Scale	TM1 (baseline)		TM2 (6 months)		TM3 (12 months)		p	p	p
	**M**	**SD**	**M**	**SD**	**M**	**SD**	**TM1 vs. TM2**	**TM1 vs. TM3**	**TM2 vs. TM3**

**COMOT-15**									

**Overall Score** **(OS)**	46,4	18,8	38,4	20,5	39,5	22,0	0,01	0,03	0,75

**Ear Symptoms** **(ES)**	35,7	18,5	27,7	18,0	28,5	20,3	0,004	0,02	0,79

**Hearing Function** **(HF)**	64,8	26,3	56,0	30,4	56,0	30,1	0,04	0,04	0,99

**Mental Health** **(MH)**	48,8	25,9	40,1	28,6	42,6	27,9	0,04	0,13	0,56

**SF-36**									

**Physical** **Functioning (PF)**	83.6	20.0	82.2	22.1	79.3	25.0	0.81	0.21	0.32

**Role-Functioning** **Physical (RP)**	74.1	39.8	76.2	38.2	73.9	37.3	0.73	0.96	0.69

**Bodily Pain** **(BP)**	72.0	27.9	73.3	26.8	74.6	26.9	0.75	0.52	0.74

**General Health** **(GH)**	58.3	19.8	59.3	18.9	56.6	19.6	0.74	0.58	0.36

**Vitality** **(VT)**	54.4	21.5	58.1	17.4	56.1	18.2	0.22	0.58	0.46

**Social Functioning** **(SF)**	77.6	25.2	79.5	22.8	78.6	22.1	0.61	0.79	0.80

**Role-Functioning** **Emotional (RE)**	75.8	37.8	78.0	38.3	73.3	38.1	0.70	0.67	0.42

**Mental Health** **(MH)**	67.0	19.6	70.1	16.6	65.4	18.4	0.26	0.59	0.08

The analysis of correlations between the scales of the COMOT-15 and the results of the audiometry showed both preoperatively and 6 months postoperatively clear associations for the scales "Hearing Function" and "Mental Health" (Table 3).

**Table 3:** Correlation analysis of the COMOT-15 scales versus PTA (air conduction) at baseline and 6 months after surgery

	PTA baseline		PTA 6 months	
**Scales of COMOT-15**	**r**	**p-value**	**r**	**p-value**

**OS**	0.24	0.02	0.36	0.0005

**ES**	-0.03	0.76	0.09	0.38

**HF**	0.43	< 0.0001	0.44	< 0.0001

**MH**	0.31	0.003	0.41	< 0.0001

PTA = pure tone average, calculated from air conduction hearing loss [dB] at 500 Hz, 1, 2 and 4 kHz (n = 90); r = Pearson's correlation coefficient. OS = Overall Score, ES = ear symptoms, HF = hearing function, MH = mental health.

Age and gender had no influence on the evaluation of the scores of COMOT-15. Furthermore, the type of CSOM (mesotympanic versus epitympanic) did not lead to different evaluations of disease-specific QOL.

Patients with revision surgery evaluated the items of the scale "Hearing Function" at all 3 time points of measurement worse compared with patients with primary surgery (TM1: p = 0.03; TM2: p = 0.006; TM3: p = 0.006). The Pearson correlation analysis between the scale "Hearing Function" and the pure tone average (PTA) for the measurement of air conduction for TM1 and TM2 revealed significant correlations (primary surgery: r = 0.44 [TM1] and r = 0.55 [TM2], revision surgery: r = 0.31 [TM1] and r = 0.29 [TM2]).

### SF-36

**Figure 1:**
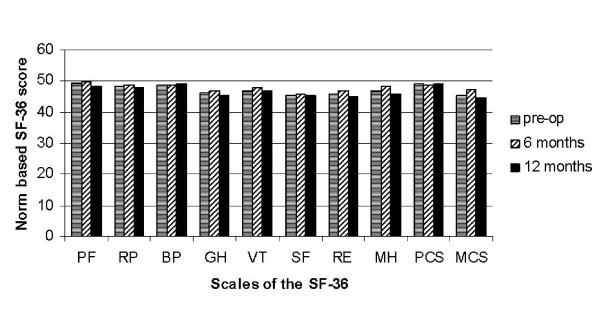
**Norm-based SF-36 scores and summary scores by time of measurement**. The scales of the SF-36: Physical Functioning (PF), Role-Functioning Physical (RP), Bodily Pain (BP), General Health (GH), Vitality (VT), Social Functioning (SF), Role-Functioning Emotional (RE), Mental Health (MH).

The evaluation of the scales of the SF-36 was not changed by the tympanoplasty (Table 2). In norm-based scoring of the SF-36, the ratings of patients were consistently slightly worse when compared with the German normal population (Figure 1).

To evaluate the influence of age on the ratings in the SF-36 data were dichotomized at the median age of 52.13 years. Older patients rated a few scales of the SF-36 better than younger patients (pre-operative: VT p = 0.01, RE p = 0.007, MH p = 0.0005; 6 months post-operatively: MH p = 0.04, 12 months after surgery: VT p = 0.01, MH p = 0.002).

Females rated the Vitality scale pre-operatively better than male patients (p = 0.02). Further gender differences were not detected.

Patients with revision surgery evaluated the items of the SF-36 similar to patients with primary surgery. Furthermore, patients with mesotympanic respectively epitympanic otitis media did not show rating differences in the scales of the SF-36.

## Discussion

Chronic suppurative otitis media (CSOM) is characterized by the clinical symptoms of hearing loss, otorrhoea, fullness of the ears, ear pain, headaches, and often tinnitus. In addition, there is usually a restriction on the ability to communicate because of the hearing loss. This often causes depression, anxiety and social withdrawal [6]. This leads to a reduced health-related QOL in different dimensions (physical, functional, social, psychological, familial) [7,8].

Health-related quality of life (HR-QOL) has an ever-increasing importance as an outcome parameter. For the proof of the success of surgical interventions, the evidence of an improvement of HR-QOL in addition to an improvement in objectively measurable parameters is required [9]. To demonstrate this evidence, the availability of validated disease-specific instruments is an essential prerequisite [10].

So far, studies on HR-QOL with validated instruments have focused on otitis media in children [11-13]. In adults, studies have been carried out with non-validated measurement tools only [14]. Other studies were focused on the influence of reduced hearing on HR-QOL, but did not pay attention to the symptoms. These studies include validated instruments like the Hearing Handicap Inventory for Adults (HHIA) and the (modified) Amsterdam Inventory Auditory Disability and Handicap Score [6,8]. Measurements of all aspects of HR-QOL in patients with CSOM with validated measurement tools were, however, to date, only rarely carried out systematically [15].

Until 2009 the Chronic Ear Survey (CES) has been the only validated instrument [16]. Evaluating the CES, we came to the opinion that the clinical symptoms of CSOM are well represented in the CES, whereas functional deficits (e.g. understanding in noisy environment) or psychological impairments (e.g. anxiety, depression) were not represented. This was for us the motivation to develop and validate the Chronic Otitis Media Outcome Test 15 (COMOT-15) [2]. In this study, the COMOT-15's suitability for the detection of disease-specific QOL in patients with CSOM has been established.

The data presented do show that patients with CSOM benefit from tympanoplasty in both the subjective and audiological evaluation. The disease-specific QOL improved in the scales "Ear Symptoms" and "Hearing Function". They stayed stable over the entire observation period, whereas the overall QOL ratings measured with the SF-36 did not indicate significant changes. Thus, the results of a study by Nadol were confirmed [1]. Disease-specific instruments have always proven to be superior to the general QOL instruments, if the disease burden was lower than the threshold measured with the general instruments [17]. Specific symptoms that may affect the conduct of life are not always sufficiently covered by the general measurement tools. Nevertheless, general instruments are essential to capture the impact of specific diseases on general health. In addition, general comparisons measuring the impact of different diseases on general QOL are possible.

The evaluation of the audiometrical studies detected a stable inner ear function, a significant mean reduction in the air bone gap by 7.9 dB and also a significant improvement in mean air conduction by 9.7 dB. Interestingly, only moderate correlations existed between the audiologically measured acoustic function and the subjectively evaluated hearing function. In other diseases it is frequently not feasible to detect correlations between objective measurements and quality of life evaluations. One example is chronic rhinosinusitis, in which the expression of the chronic inflammatory changes in computed tomography of the sinuses is not correlated with the subjectively evaluated symptoms [18].

The type of CSOM (mesotympanic versus epitympanic) had no influence on the evaluation of disease-specific QOL. This result is for the clinically active otologists initially surprising, since the genesis of the two different types of CSOM could have been anticipated by the patient differently. The course of untreated epitympanal CSOM is more difficult and causes more serious complications complicated than the course of mesotympanal CSOM. Surely early recognition and treatment of both types of CSOM was ensuring that these potential differences did not manifest in our study cohort. In this context, the worse evaluation of subjective QOL by patients with revision surgery as compared to the primary surgery patients can possibly be explained by the prolonged course and associated higher burden of the disease.

## Conclusions

Tympanoplasty did lead to a significant improvement of disease-specific HR-QOL in patients with CSOM while general HR-QOL did not change. Very well correlations were found between the subscale hearing function from the COMOT-15 questionnaire and audiological findings. Revision surgery seems to be a predictor for a worse outcome.

## Competing interests

The authors declare that they have no competing interests.

## Authors' contributions

IB conceived of the study, and participated in its design and coordination and helped to draft the manuscript. BG monitored data collection, participated in the design and coordination of the study and helped to draft the manuscript. PKP participated in drafting the script. MP participated in its design and coordination and helped to draft the manuscript. All authors read and approved the final manuscript.

## Supplementary Material

Additional File 1 **Chronic Otitis Media Outcome Test 15 (COMOT-15)**. Click here for file
