# Supplementary figures and images for: General and disease-specific quality of life in patients with chronic suppurative otitis media - a prospective study
Source: Health Qual Life Outcomes. 2011 Jun 29;9:48. doi: 10.1186/1477-7525-9-48 (PMC3148957; doi:10.1186/1477-7525-9-48)

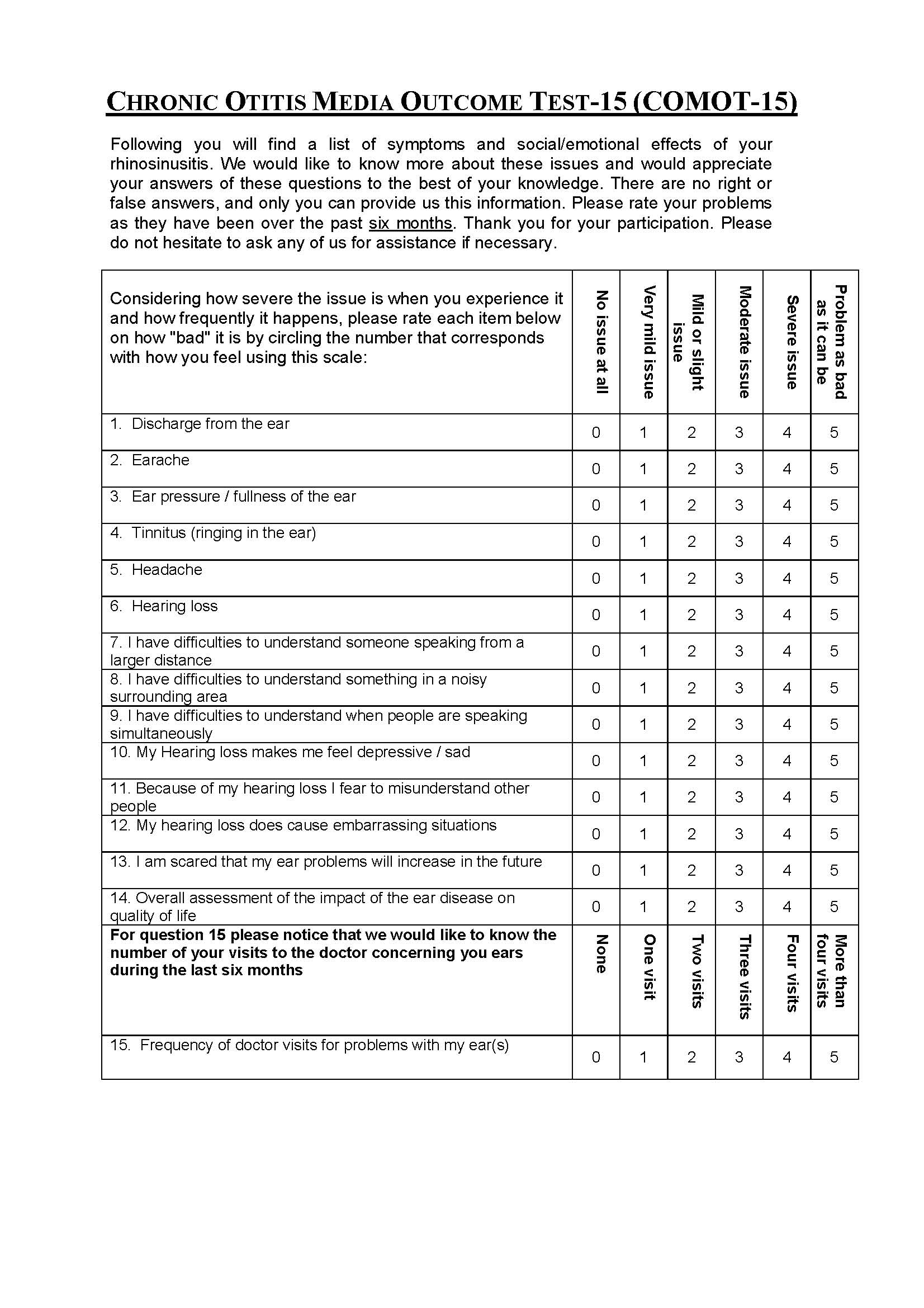

Supplement: Additional File 1 — Chronic Otitis Media Outcome Test 15 (COMOT-15). [file 1477-7525-9-48-S1.JPEG]
